# Protein acetylation in atherosclerosis: beyond inflammation to core cellular processes and therapeutic potential

**DOI:** 10.3389/fimmu.2026.1784874

**Published:** 2026-07-28

**Authors:** Zhaoyang Dong, Yingli Zhou, Yuxuan Gao, Rui Guo, Xuan He, Yating Deng, Yuhua Chen, Qianhui Zhu, Jiaming Wei, Zhihua Guo

**Affiliations:** 1 College of Chinese Medicine, Hunan University of Chinese Medicine, Changsha, Hunan, China; 2 Institute of Traditional Chinese Medicine (TCM) Diagnostics, Hunan University of Chinese Medicine, Changsha, Hunan, China; 3 Provincial Key Laboratory of Traditional Chinese Medicine (TCM) Diagnostics, Hunan University of Chinese Medicine, Changsha, Hunan, China; 4 First Clinical College of Chinese Medicine, Hunan University of Chinese Medicine, Changsha, Hunan, China; 5 Changsha Hospital of Traditional Chinese Medicine (Changsha Eight Hospital), Changsha, Hunan, China; 6 College of Traditional Chinese Medicine, Changsha Medical University, Changsha, Hunan, China

**Keywords:** atherosclerosis, inflammatory response, protein acetylation, protein acetyltransferases, protein deacetylases

## Abstract

Atherosclerosis (AS) is the leading cause of cardiovascular disease-related mortality worldwide and serves as the core pathological basis for cardiovascular events. Protein acetylation, a widespread and highly dynamic post-translational modification, has emerged as a critical link connecting epigenetic regulation, metabolic homeostasis, and inflammatory signaling, thereby playing an important role in both the initiation and progression of AS. This review systematically summarizes the major forms of protein acetylation, including N-terminal acetylation and lysine acetylation, as well as the key regulatory enzymes involved, such as acetyltransferases (e.g., HATs and NATs) and deacetylases (e.g., HDACs and sirtuins). Particular emphasis is placed on the cell type-specific regulatory roles of acetylation in macrophages, vascular endothelial cells, and vascular smooth muscle cells. Accumulating evidence indicates that protein acetylation modulates gene transcription and protein function through multiple mechanisms, thereby influencing a broad spectrum of AS-related processes, including inflammation, glycolipid metabolism, oxidative stress, energy metabolism, apoptosis, proliferation, and migration. Based on these mechanisms, therapeutic strategies targeting enzymes that regulate acetylation, particularly selective HDAC inhibitors and sirtuin activators, have emerged as promising approaches for the treatment of AS. By integrating recent advances in cellular heterogeneity, plaque stage-specific regulation, and human translational evidence, this review further discusses the therapeutic potential of targeting acetylation-regulating enzymes and critically evaluates the current limitations of this strategy, including contradictory findings, off-target effects, and barriers to clinical translation. Overall, protein acetylation represents a key regulatory hub linking epigenetics, metabolism, and inflammation. A deeper understanding of its regulatory network may provide new insights into the development of precision therapies for AS.

## Graphical Abstract

**Figure:**
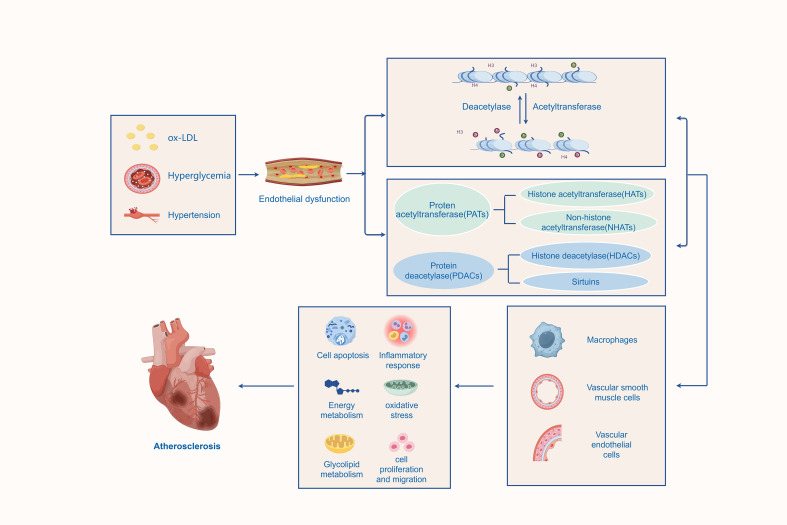

## 1 Introduction

Atherosclerosis (AS) is a chronic inflammatory disease characterized by lipid deposition in the arterial wall and is a leading cause of cardiovascular morbidity and mortality (1). Its pathogenesis involves a series of interrelated processes, including endothelial dysfunction, lipid accumulation, inflammatory cell infiltration, smooth muscle cell proliferation, and extracellular matrix (ECM) remodeling, which collectively drive disease initiation and progression (2, 3). Pathological changes are initiated when large amounts of low-density lipoprotein (LDL) undergo oxidative modification by reactive oxygen species (ROS), generating oxidized low-density lipoprotein (ox-LDL), a highly pro-inflammatory and immunogenic molecule. Ox-LDL accumulates beneath the vascular endothelium, particularly at arterial branch points and curvatures exposed to disturbed flow and low shear stress (4). This subendothelial retention of ox-LDL promotes the recruitment and adhesion of circulating monocytes and lymphocytes, facilitating their infiltration into the arterial wall. The progressive accumulation of inflammatory cells leads to intimal thickening and foam cell formation, thereby contributing to AS development (5, 6). As the disease progresses, the persistent inflammatory microenvironment induces smooth muscle cell apoptosis, senescence, and phenotypic switching. Fibrous plaques gradually develop into complex lesions containing a fibrous cap and necrotic core. Although the fibrous cap contributes to plaque stability, expansion of the necrotic core is closely associated with plaque vulnerability and progression (7, 8).

Post-translational modifications (PTMs) are covalent modifications that occur on one or more amino acid residues during or after protein translation (9). Among these modifications, protein acetylation has emerged as a key regulatory mechanism. Acetylation is catalyzed by acetyltransferases, which transfer acetyl groups from donor molecules to specific residues on target proteins. By modulating chromatin structure, transcription, and signal transduction, acetylation regulates cell cycle progression, metabolism, and other essential cellular processes (10).

In recent years, protein acetylation, a widespread and highly dynamic PTM, has been increasingly recognized as a key regulator of multiple pathophysiological processes in AS through its precise control of gene transcription and protein function (11). This review summarizes the major classes and key enzymatic systems involved in protein acetylation, with particular emphasis on acetylation-dependent regulatory networks in major AS-related cell types, including macrophages, vascular endothelial cells, and vascular smooth muscle cells (VSMCs). By modulating inflammation, glycolipid metabolism, oxidative stress, energy metabolism, apoptosis, proliferation, and migration, protein acetylation contributes to both the initiation and progression of AS. In addition, this review incorporates emerging concepts of cellular heterogeneity, plaque stage-specific regulation, and human clinical evidence, thereby moving beyond the limitations of the traditional M1/M2 polarization paradigm. Furthermore, the therapeutic potential of targeting acetylation-modifying enzymes, such as histone acetyltransferases (HATs), histone deacetylases (HDACs), and sirtuins, as well as the challenges associated with their clinical translation, are also discussed. Overall, this review provides a systematic framework for understanding the epigenetic regulatory mechanisms underlying AS and may offer insight into the development of precision therapeutic strategies.

## 2 Overview of protein acetylation

Protein acetylation is a widespread and highly conserved PTM in which an acetyl group (-COCH3) is added to specific protein moieties, thereby regulating diverse biological processes in eukaryotic cells (12). Protein biosynthesis begins with the translation of mRNA into a polypeptide chain, followed by the transport of the nascent proteins to distinct cellular compartments. During this process, proteins undergo various PTMs, which greatly expand their structural complexity and functional diversity. Acetylation can occur on multiple chemical groups, including hydroxyl, sulfhydryl, and amino groups. It was first identified in nuclear histones, but subsequent studies have shown that acetylation is also widely distributed in non-histone proteins, where it regulates cellular responses to intracellular and extracellular stimuli as well as signal transduction (12). Although hundreds of PTMs have been identified in the eukaryotic proteome, only a limited number have been characterized in detail (13, 14).

Protein acetylation is a dynamic and reversible process in which acetyl groups are covalently added to specific amino acid residues by acetyltransferases and removed by deacetylases, forming an important regulatory mechanism. From a functional perspective, protein acetylation can be broadly classified into histone acetylation and non-histone acetylation. Based on the acetylation site, it is mainly categorized into N-terminal α-amino acetylation, lysine ϵ-amino acetylation, and acetylation on other amino acid side chains (15). Increasing evidence suggests that PTMs are critically involved in the pathogenesis of multiple diseases, including obesity, cancer, non-alcoholic fatty liver disease (NAFLD), and type 2 diabetes (T2D) (16).

### 2.1 Protein N-terminal acetylation (N-terminal α-amino acetylation)

N-terminal α-amino acetylation is widely conserved across species and represents one of the most common protein modifications in eukaryotic cells (17). The modification involves the transfer of an acetyl group from acetyl coenzyme A (acetyl-CoA) to the N-terminal α-amino group of a nascent peptide chain, a reaction catalyzed by N-terminal acetyltransferases (NATs) (18). NATs constitute a family of enzymes localized to specific cellular compartments and regulate protein stability, protein–protein interactions, and biological function through N-terminal acetylation. Among cytosolic NATs, NatA primarily acetylates small amino acid residues exposed after removal of the initiator methionine (Met). Through mechanisms such as the Ac/N-end rule pathway, NatA regulates protein stability and protein–protein interactions (19), contributing to stress responses and cell cycle regulation (20). NatB acetylates proteins that retain the initiator Met followed by an acidic residue and plays important roles in actin cytoskeleton organization and DNA damage repair (21). Other cytosolic and nuclear NATs, including NatC, NatD, NatE, and NatF, recognize broader classes of N-terminal sequences. Among these enzymes, NatF preferentially acetylates proteins with an N-terminal Met followed by hydrophobic residues and regulates the subcellular localization of proteins associated with the Golgi apparatus and plasma membrane (22). In contrast, NatD exhibits high substrate specificity and selectively acetylates the N-termini of histones H4 and H2A (23).

### 2.2 Protein lysine acetylation

Lysine acetylation is catalyzed by lysine acetyltransferases (KATs), which transfer an acetyl group from acetyl-CoA to the ϵ-amino side chain of lysine residues (24). The modification is tightly regulated by two opposing enzyme families: HATs, which catalyze acetyl group addition, and HDACs, which mediate acetyl group removal (25). Lysine acetylation was initially associated with chromatin remodeling and transcriptional regulation. However, the discovery of numerous non-histone substrates, including transcription factors, metabolic enzymes, and cytoskeletal proteins, has substantially expanded its recognized functional scope, indicating its broad involvement in metabolism, cell proliferation, cellular excitability, and muscle contraction and relaxation (26). In histones, acetylation at specific lysine residues (e.g., H3K9, H3K14, H3K27, H4K16) is a key component of the histone code (25, 27). By neutralizing the positive charge of lysine residues, acetylation weakens histone–DNA interactions and promotes the recruitment of bromodomain-containing transcriptional activators, thereby increasing chromatin accessibility and facilitating transcription (28, 29). In non-histone proteins, lysine acetylation also exerts broad regulatory effects. It modulates the DNA-binding activity, stability, and transcriptional function of proteins such as p53 (30), NF-κB (31), and STAT3 (32), and also affects signaling enzymes, including ataxia telangiectasia mutated (ATM) (33) and AMP-activated protein kinase (AMPK) (34).

### 2.3 Acetylation modifications of other amino acid side chains

In addition to N-terminal and lysine acetylation, several other amino acid side chains can also undergo acetylation. Among these, O-acetylation of serine and threonine has been observed predominantly in bacteria (35) and has also been reported in eukaryotic systems (36), although its functional significance remains poorly defined. Arginine N-acetylation is relatively rare, but emerging evidence suggests that it may antagonize methylation, thereby influencing protein–protein interactions (37). Cysteine S-acetylation has been implicated in metabolic regulation in bacteria, whereas its physiological relevance in mammalian cells remains largely unclear (38, 39).

## 3 Key enzymes in protein acetylation

Protein acetylation is regulated by a coordinated network of acetyltransferases and deacetylases that control essential cellular processes, including gene expression, signal transduction, and metabolic homeostasis (40, 41). Through the dynamic addition and removal of acetyl groups, these enzymes modulate protein activity, stability, and subcellular localization, thereby forming a fundamental regulatory mechanism for cellular function (42).

In this regulatory system, protein acetyltransferases (PATs) catalyze the transfer of acetyl groups from acetyl-CoA to specific residues on target proteins (43). According to substrate preference, PATs can be broadly classified into two groups: HATs, which predominantly target histones, and non-histone acetyltransferases with broader substrate specificity (44). HATs mainly act on nuclear histones, promote chromatin relaxation by neutralizing lysine positive charges, and facilitate transcriptional activation (45, 46). Members of the GCN5-related N-acetyltransferase (GNAT) family, including GCN5 and p300/CBP-associated factor (PCAF), acetylate specific lysine residues on histone H3 and thereby enhance gene expression (47). TIP60, a member of the MYST family, contributes to DNA damage repair and apoptosis by acetylating histone H4 and tumor suppressor p53 (48). The p300/CBP family functions as a central regulatory complex that acetylates histones and transcription factors such as p53 and NF-κB, thereby influencing cell proliferation, differentiation, and stress responses (49). In contrast, non-histone acetyltransferases are mainly distributed in the cytoplasm and mitochondria. Mitochondrial acetyltransferases, such as acetyl-CoA acetyltransferase 1 (ACAT1) and GCN5-like protein 1 (GCN5L1), regulate the activity of key metabolic enzymes, whereas members of the NAT family contribute to protein folding and subcellular localization through N-terminal acetylation, thereby supporting metabolic homeostasis and structural stability (46).

Protein deacetylases (PDACs) function as counterparts to acetyltransferases and maintain acetylation balance by removing acetyl groups from proteins. Deacetylases are broadly classified into two groups: the classical Zn2+-dependent HDACs (classes I, II, IV) and the NAD+-dependent Sirtuin family (class III HDACs) (50, 51). Classical HDACs require Zn2+ as a catalytic cofactor. Class I HDACs (e.g., HDAC1-3, 8) are predominantly localized in the nucleus, where they associate with transcriptional repression complexes to promote chromatin condensation and suppress gene expression, thereby contributing to cell cycle regulation. Class II HDACs shuttle between the nucleus and cytoplasm and exert more diverse regulatory effects. Among them, HDAC6 is mainly cytoplasmic and deacetylates substrates such as α-tubulin and heat shock proteins, thereby regulating cytoskeletal remodeling and protein quality control (52–54). Class IV HDAC11 has been implicated in the regulation of immune-related genes and immune responses. In contrast, the sirtuin family depends on NAD+ and exhibits broader functional diversity (55). SIRT1 is mainly localized in the nucleus and regulates genes involved in aging and metabolic homeostasis, whereas SIRT2 is primarily cytoplasmic and participates in metabolic regulation and cell cycle progression. Mitochondrial SIRT3, SIRT4, and SIRT5 contribute to energy metabolism and antioxidant defense. SIRT6 supports DNA repair and telomere maintenance, and its deficiency has been associated with premature aging. SIRT7 is predominantly localized in the nucleolus, where it regulates ribosome biogenesis and cellular stress responses (56).

## 4 The role of protein acetylation in AS and related major cells

### 4.1 Inflammatory response

**Figure 1.**

**Figure 1:**
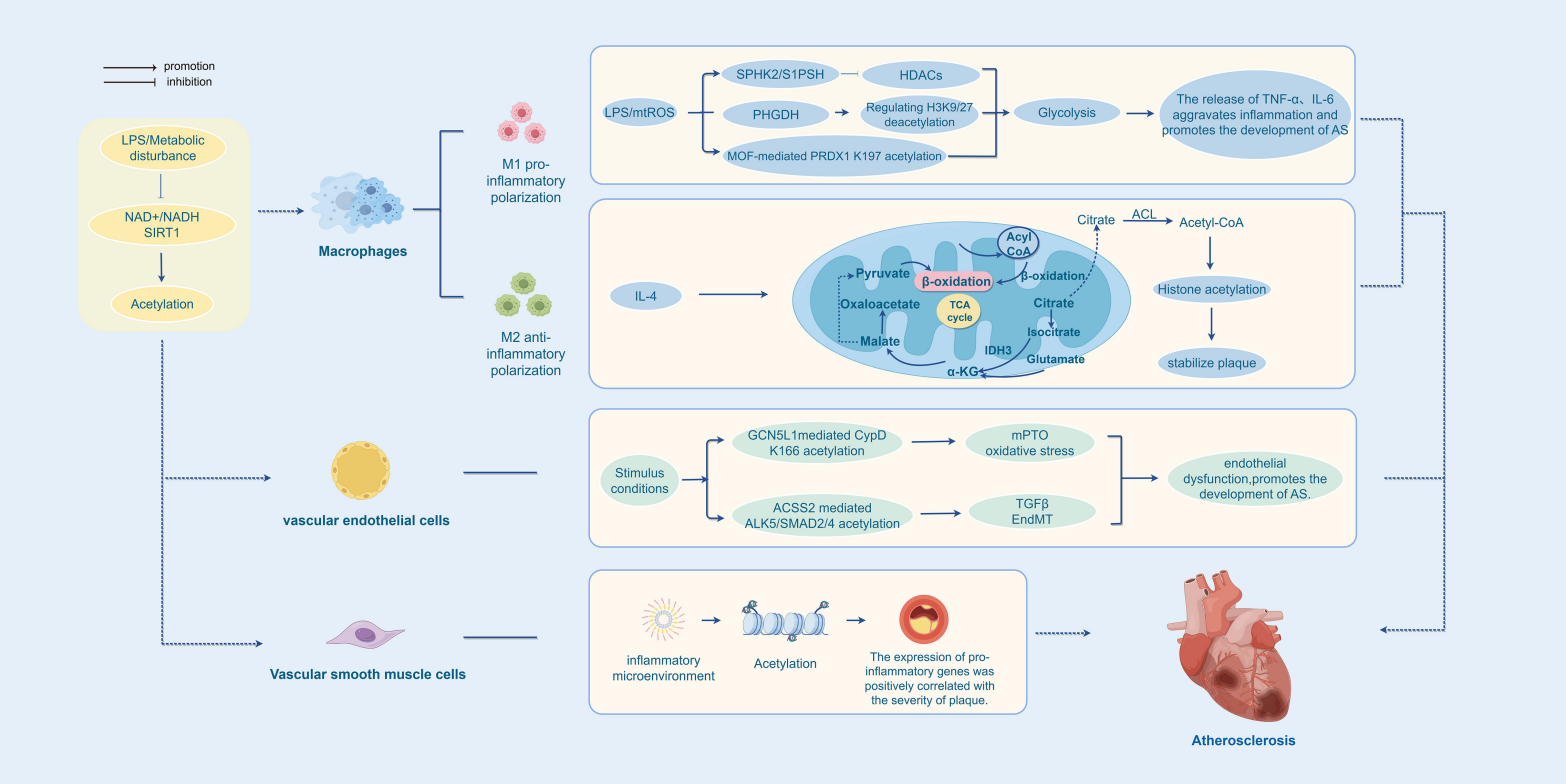
Inflammatory response.

The pathogenesis of AS involves the interplay of multiple processes, including endothelial dysfunction, lipid accumulation, and inflammatory mediator production (57). Inflammation persists throughout both the initiation and progression of AS, promoting VSMC proliferation and migration, disturbing the balance between ECM synthesis and degradation, and thereby driving plaque formation and growth (58). In addition, inflammation critically affects plaque stability, as proteases and other mediators released by inflammatory cells degrade the fibrous cap, thereby increasing the risk of plaque rupture and triggering acute cardiovascular events (59).

NF-κB, composed of homodimers or heterodimers of five subunits (p50, p52, RelA/p65, RelB, and c-Rel), is a central transcriptional regulator of AS-associated inflammation (60). Its activity is tightly regulated by acetylation (61). Acetylation of the p65 subunit at lysine 310 (K310) is required for full transcriptional activation of NF-κB (62). Lipopolysaccharide (LPS) reduces the intracellular NAD+/NADH ratio and suppresses the activity of the NAD+-dependent deacetylase SIRT1, resulting in increased p65 K310 acetylation and enhanced NF-κB activation. In contrast, glycolytic inhibition can restore NAD+ levels, reactivate SIRT1, promote p65 deacetylation, and suppress NF-κB-dependent expression of pro-inflammatory mediators (such as TNF-α, IL-6, iNOS, etc.) (61). Notably, the pathological relevance of this pro-inflammatory acetylation pattern is also supported by human evidence. In advanced human atherosclerotic plaques, p300 is upregulated and colocalizes with inflammatory regions, whereas the protective deacetylases SIRT1 and SIRT6 are frequently downregulated (63–65). These findings suggest that disruption of the acetylation–deacetylation balance is a common feature of inflammation in human AS.

Macrophages serve as central components of innate immunity and participate in multiple phases of the inflammatory response. As highly plastic cells, they adopt distinct functional states in response to local microenvironmental cues (63, 64). In the traditional framework, macrophages are broadly classified into M1 and M2 phenotypes. M1 macrophages primarily rely on glycolysis and exert pro-inflammatory effects. They accumulate in early lesions and release cytokines such as TNF-α, IL-1, and IL-6, thereby amplifying inflammation and promoting AS progression (65). In contrast, M2 macrophages rely on fatty acid oxidation and exert anti-inflammatory and tissue-reparative effects. In advanced lesions, they facilitate the clearance of apoptotic cells and necrotic debris, thereby contributing to plaque stabilization and limiting disease progression (64, 66). Histone modifications, including acetylation, methylation, phosphorylation, ubiquitination, and ADP-ribosylation, represent major epigenetic mechanisms regulating macrophage phenotype (67). Multiple studies have shown that these modifications play important roles in macrophage functional reprogramming (68–70). However, although the M1/M2 polarization model provides a useful conceptual framework, recent single-cell transcriptomic studies indicate that plaque macrophages exist along a broad continuum of functional states rather than within a simple binary classification (71). These states include proliferative, inflammatory, oxidative stress-responsive, and efferocytic subsets, and their dynamic changes are closely associated with plaque progression and stability. Therefore, although the following discussion refers to the classical M1/M2 paradigm, it also incorporates emerging concepts such as macrophage heterogeneity and plaque stage-specific regulation, providing a more precise framework for understanding how acetylation modulates AS-associated inflammation.

Macrophage polarization refers to the acquisition of distinct functional states in response to specific stimuli. Protein acetylation plays a critical regulatory role in this process by modulating signaling pathways, cellular metabolism, and gene expression. Evidence indicates that LPS induces pro-inflammatory polarization, whereas IL-4 or IL-10 drives anti-inflammatory polarization. During M1 polarization, MOF-mediated acetylation of peroxiredoxin 1 (PRDX1) at lysine 197 (K197) has been shown to be functionally important. Following LPS stimulation, alterations in PRDX1 acetylation regulate intracellular hydrogen peroxide accumulation, extracellular signal-regulated kinase 1/2 (ERK1/2) phosphorylation, glycolytic reprogramming, and IL-6 production, thereby shaping macrophage activation (72). In addition, phosphoglycerate dehydrogenase (PHGDH) deficiency promotes deacetylation of H3K9 and H3K27, enhances NAD+-dependent deacetylase activity, and suppresses Tlr4 transcription. PHGDH deficiency also alters both histone and non-histone acetylation, while SIRT2-mediated regulation of NLRP3 acetylation further affects inflammasome activation and contributes to M1 polarization (73). Consistently, sphingosine kinase 2 (SPHK2) activity, NLRP3 inflammasome activation, and mitochondrial ROS levels are markedly increased in LPS-treated macrophages. SPHK2 knockdown or pharmacological inhibition attenuates LPS-induced M1 polarization, oxidative stress, and NLRP3 activation. Mechanistically, increased SPHK2 elevates nuclear sphingosine-1-phosphate (S1P), which restrains HDAC activity and promotes p53 acetylation (67).

Protein acetylation also plays an important role in M2 macrophage polarization. Evidence indicates (74) that IL-4-induced polarization from M0 to M2 macrophages is accompanied by increased tricarboxylic acid (TCA) cycle activity, which directly supports histone acetylation. Inhibition of mitochondrial pyruvate carrier 1 or deficiency of ATP-citrate lyase (ACLY) impairs histone acetylation and M2 polarization, whereas exogenous acetate largely reverses these effects, highlighting the importance of metabolic control in acetylation-dependent macrophage reprogramming. ACLY acts as a key link between mitochondrial metabolism and epigenetic regulation by converting TCA cycle-derived citrate into acetyl-CoA, thereby sustaining the nuclear–cytoplasmic acetyl-CoA pool required for histone acetylation (71). Although most current evidence for acetylation-regulated macrophage polarization is derived from inflammatory and metabolic studies, findings in tumor-associated macrophages further support the broader relevance of this mechanism. For example, mutations in CREBBP and EP300 may influence macrophage polarization through the FBXW7–NOTCH–CCL2/CSF1 axis, suggesting that dysregulated histone acetylation can reshape the local immune microenvironment (75).

Protein acetylation also regulates the inflammatory response in endothelial cells and modulates the activation of inflammatory signaling pathways. Inflammation can be classified as acute or chronic (76), and chronic inflammation underlies multiple disorders, including allergies, AS, and neurodegenerative diseases (77). In endothelial cells, GCN5L1-mediated acetylation of mitochondrial cyclophilin D (CypD) at K166 promotes mitochondrial permeability transition pore (mPTP) opening, increases oxidative stress, induces a maladaptive metabolic shift toward glycolysis, and suppresses endothelial nitric oxide synthase (eNOS) activity through disruption of the GCN5L1/SIRT3 balance. These changes contribute to endothelial dysfunction and hypertension, whereas inhibition of this acetylation event ameliorates vascular injury (78). Additional studies have shown that activation of TGFβ signaling reduces PDK4 expression in endothelial cells, enhances pyruvate dehydrogenase (PDH) activity, and abnormally increases acetate production. Acetate is converted to acetyl-CoA by ACSS2, which acetylates the TGFβ receptor ALK5 and SMAD2/SMAD4, thereby strengthening and stabilizing TGFβ signaling and promoting endothelial-to-mesenchymal transition (EndMT). Endothelial-specific ACSS2 deletion reduces this acetylation and mitigates atherosclerotic lesion development (79). Notably, the inflammatory effects of protein acetylation appear to be stage-dependent during plaque progression. In early AS, the anti-inflammatory cytokine IL-35 alleviates endothelial activation by suppressing H3K14 acetylation (80). In contrast, in advanced plaques, acetylation levels of H3K9 and H3K27 are markedly increased in VSMCs and macrophages and are positively associated with plaque severity (81, 82). This transition from early protective suppression to late-stage pathological acetylation highlights the importance of defining an appropriate therapeutic window for acetylation-targeted intervention.

In summary, protein acetylation is a central regulator of AS-associated inflammation. On one hand, acetylation modulates the activity of the key transcription factor NF-κB, thereby influencing the expression of pro-inflammatory factors. On the other hand, it participates in multiple stages of AS initiation and progression by regulating macrophage polarization, endothelial function, and metabolic reprogramming. The balance between acetylation and deacetylation shows marked temporal and spatial specificity during inflammatory regulation, with distinct modification patterns observed between early and advanced plaques. This dynamic imbalance may serve not only as an indicator of inflammatory status but also as a potential driver of disease progression. However, current studies on acetylation in AS remain limited by several factors, including an excessive focus on individual cell types, an incomplete understanding of the regulatory networks of acetylation-modifying enzymes and their crosstalk with other post-translational modifications, continued reliance on the traditional macrophage polarization model, and a lack of dynamic analyses linking single-cell profiles to plaque stage. In addition, the specificity, translational potential, and safety of acetylation-targeted therapies require further validation. Future studies should integrate multi-omics approaches, single-cell lineage tracing, and tissue-specific gene editing to systematically clarify the dynamic regulatory mechanisms of acetylation and advance precision therapeutic strategies for AS.

### 4.2 Glycolipid metabolism.

**Figure 2.**

**Figure 2:**
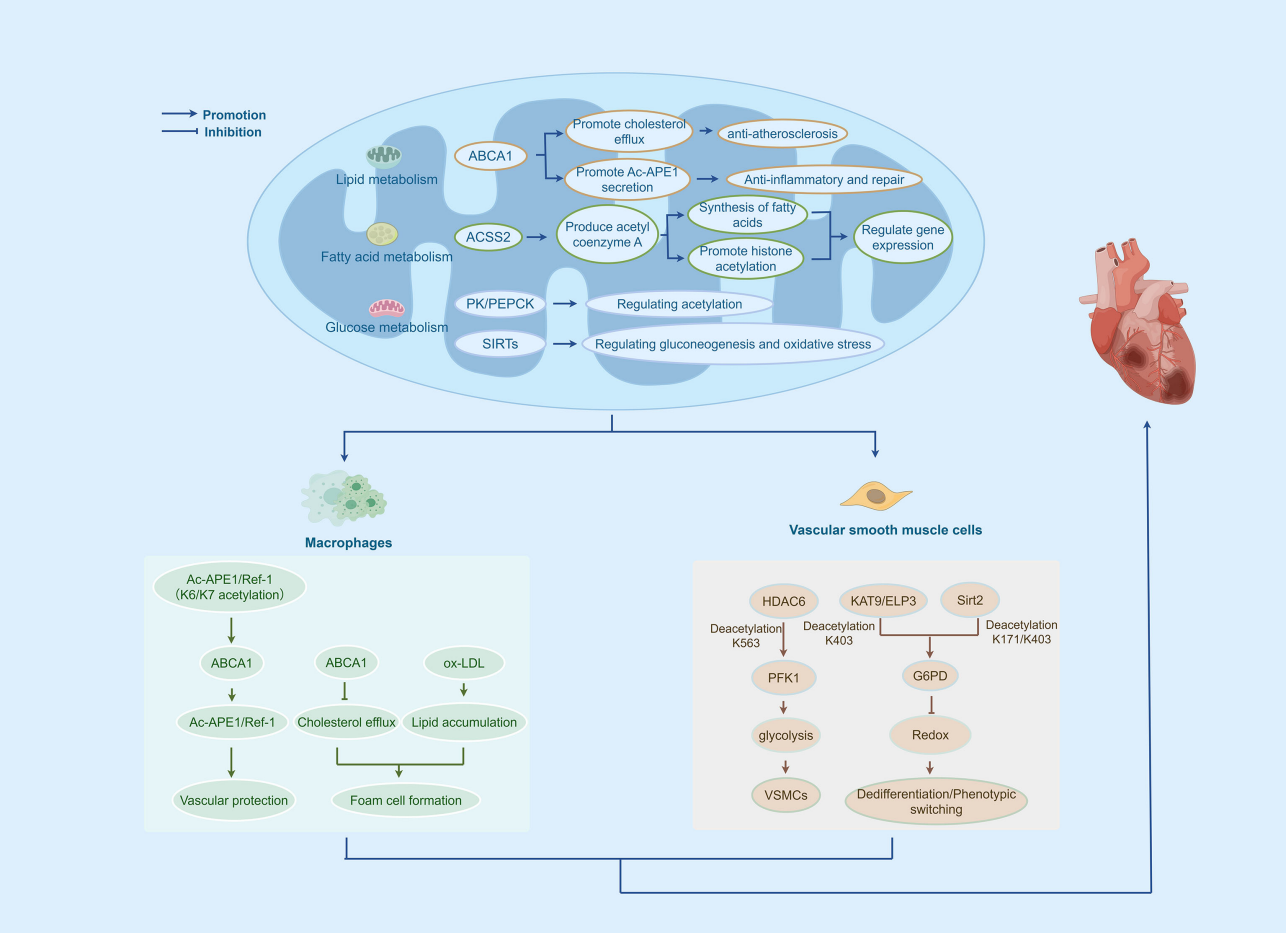
Glucose and lipid metabolism.

Glycolipid metabolism is a major source of cellular energy and is essential for maintaining normal physiological function in tissues and organs. Dysregulation of this metabolic network contributes to the development of AS (83), obesity (84), and cancer (85). During AS initiation and progression, protein acetylation acts as an important post-translational regulatory mechanism by modulating key pathways involved in glucose and lipid metabolism.

Reverse cholesterol transport (RCT) is a central mechanism for maintaining cholesterol homeostasis, and ATP-binding cassette transporter A1 (ABCA1) is a critical mediator of this pathway. ABCA1 promotes the efflux of intracellular cholesterol and phospholipids to apolipoprotein A-I, thereby initiating nascent high-density lipoprotein (HDL) formation (80). Studies further indicate (81, 82) that ABCA1 acts not only as a central regulator of cholesterol efflux but also as a binding partner for acetylated APE1/Ref-1 (AcAPE1/Ref-1), thereby facilitating its secretion. Acetylation of APE1/Ref-1 (e.g., at K6/K7 sites) is required for its secretion and directly affects its colocalization with ABCA1 and secretion efficiency (86). Secreted AcAPE1/Ref-1 exerts vasculoprotective effects through anti-inflammatory activity and DNA damage repair, thereby attenuating vascular injury associated with lipid metabolic imbalance.

Acetylation plays a crucial regulatory role in fatty acid metabolism (87). Acyl-CoA short-chain synthetases (ACSSs) are key enzymes that convert short-chain fatty acids into acetyl-CoA and are distributed in both the cytoplasm and nucleus (88, 89). Among them, ACSS2 exhibits context-dependent functions according to nutrient availability and cellular stress. Under nutrient-replete and unstressed conditions, ACSS2 mainly supports cytoplasmic metabolism by generating acetyl-CoA for fatty acid synthesis, thereby promoting lipid synthesis and storage. In contrast, under nutrient deprivation, stress, or tissue injury, ACSS2 translocates to the nucleus, where it cooperates with transcriptional complexes to regulate histone acetylation and gene expression through local acetyl-CoA production (90, 91). Evidence further suggests that ACSS2 not only dynamically regulates acetylation at specific histone sites but also influences global histone acetylation levels through its control of acetyl-CoA availability in both cytoplasmic and nuclear compartments (92). As a key metabolic intermediate and signaling molecule, acetyl-CoA supports fatty acid synthesis while simultaneously serving as the acetyl donor for histone acetylation, thereby linking lipid metabolism to epigenetic regulation (43, 93). Increased nuclear acetyl-CoA levels may enhance KAT activity and promote histone acetylation (94).

Glycolysis degrades glucose to generate energy and biosynthetic intermediates, whereas gluconeogenesis produces glucose to maintain blood glucose homeostasis during starvation (95). Although these pathways share several enzymes, they are not simple reversals of one another and are instead independently and reciprocally regulated. Key enzymes involved in their irreversible steps, including pyruvate kinase (PK) and phosphoenolpyruvate carboxykinase (PEPCK), are subject to lysine acetylation. Increasing evidence indicates that sirtuin-dependent deacetylation is an important regulator of glucose metabolism. For example, SIRT2 improves impaired hepatic glucose uptake by deacetylating glucokinase regulatory protein (GKRP) at lysine 126 (96). SIRT1 promotes gluconeogenesis through deacetylation of peroxisome proliferator-activated receptor gamma coactivator-1α (PGC-1α), whereas SIRT6 suppresses gluconeogenesis by promoting PGC-1α acetylation in an acetyltransferase 5-dependent manner (97, 98). In addition, reduced SIRT3 expression exacerbates oxidative stress and apoptosis in diabetic mouse hearts by decreasing the deacetylation of superoxide dismutase 2 (SOD2) (99).

Macrophages are the most abundant immune cells in atherosclerotic plaques and play essential roles in plaque initiation, progression, and rupture (100). Acetylation of proteins involved in cholesterol metabolism substantially affects cholesterol uptake, efflux, and intracellular homeostasis by regulating key molecules such as scavenger receptors (e.g., CD36) and ABCA1. When macrophages internalize more lipoprotein-derived cholesterol than they can efflux, excess free cholesterol is esterified and stored in lipid droplets, leading to foam cell formation, a hallmark of early atherosclerotic lesions (101). Hypercholesterolemia and related metabolic disturbances also stimulate bone marrow monocyte production, resulting in monocytosis, an independent risk factor for atherosclerotic disease (5, 102, 103). These circulating monocytes adhere to dysfunctional endothelium, infiltrate arterial regions exposed to disturbed flow, and differentiate into lesional macrophages (104). During early lesion development, macrophages accumulate in the intima, engulf modified lipids, and secrete chemokines that recruit additional monocytes, thereby promoting lesion progression. In advanced plaques, increased macrophage apoptosis together with defective efferocytosis amplifies inflammation, enlarges the necrotic core, and promotes fibrous cap thinning, thereby increasing plaque vulnerability. In contrast, during plaque regression, macrophage abundance declines and their transcriptional profile shifts toward a pro-resolving phenotype, which can be induced by intensive lipid-lowering or glycemic control (101). Macrophages also internalize ox-LDL through scavenger receptors while downregulating cholesterol efflux-related genes, including ABCA1 and ATP-binding cassette transporter G1 (ABCG1), which together drive foam cell formation (79).

VSMCs are the principal cellular component of the tunica media (105). Glucose-6-phosphate dehydrogenase (G6PD) is a critical regulator of VSMC function and is closely associated with smooth muscle phenotypic modulation. Inhibition or knockdown of G6PD promotes the expression of smooth muscle-specific genes, preserves the differentiated phenotype, and limits vascular remodeling, whereas G6PD overexpression favors dedifferentiation and may contribute to atherosclerotic progression (106–108). Acetylation is an important post-translational mechanism regulating G6PD and is coordinated by acetyltransferases and deacetylases (109). Through its role in nicotinamide adenine dinucleotide phosphate (NADPH) production, G6PD supports redox homeostasis and metabolic balance, whereas acetylation at lysine 171 and lysine 403 (K171 and K403) modulates its enzymatic activity and affects VSMC function (110). The acetyltransferase KAT9/ELP3 catalyzes acetylation at K403 and inhibits G6PD activity, whereas SIRT2 enhances G6PD activity and maintains redox balance by deacetylating K171 and K403 (111). Elevated Nesfatin-1 expression has been observed in calcified VSMCs, aortic tissues, and patients with coronary artery calcification (112). Phosphofructokinase 1 (PFK1), a rate-limiting enzyme in glycolysis, is also regulated by acetylation (113). Studies have shown that HDAC6 mediates deacetylation of PFK1 at K563 in PDGF-BB-stimulated proliferating VSMCs. Deacetylation at K563 promotes assembly of PFK1 monomers into more active tetramers, thereby enhancing its enzymatic activity. Increased PFK1 activity accelerates glycolysis, increases energy and metabolic intermediate production, and promotes VSMC proliferation and growth (114, 115).

Overall, protein acetylation is a central regulator of glycolipid metabolism in AS. By modulating the activity, localization, and functional interactions of key molecules such as ABCA1, ACSS2, PGC-1α, G6PD, and PFK1, acetylation influences multiple pathological processes, including macrophage foam cell formation and VSMC proliferation. However, current studies remain limited in several respects. Most have focused on individual proteins or specific acetylation sites, with insufficient integration of the broader acetylation regulatory network. In addition, the spatiotemporal dynamics of acetylation under pathological conditions remain poorly understood, and the influence of cellular heterogeneity and microenvironmental context has often been overlooked. Moreover, precision therapeutic strategies targeting acetylation are still at an early stage, posing substantial challenges for clinical translation.

### 4.3 Oxidative stress

**Figure 3**.

**Figure 3:**
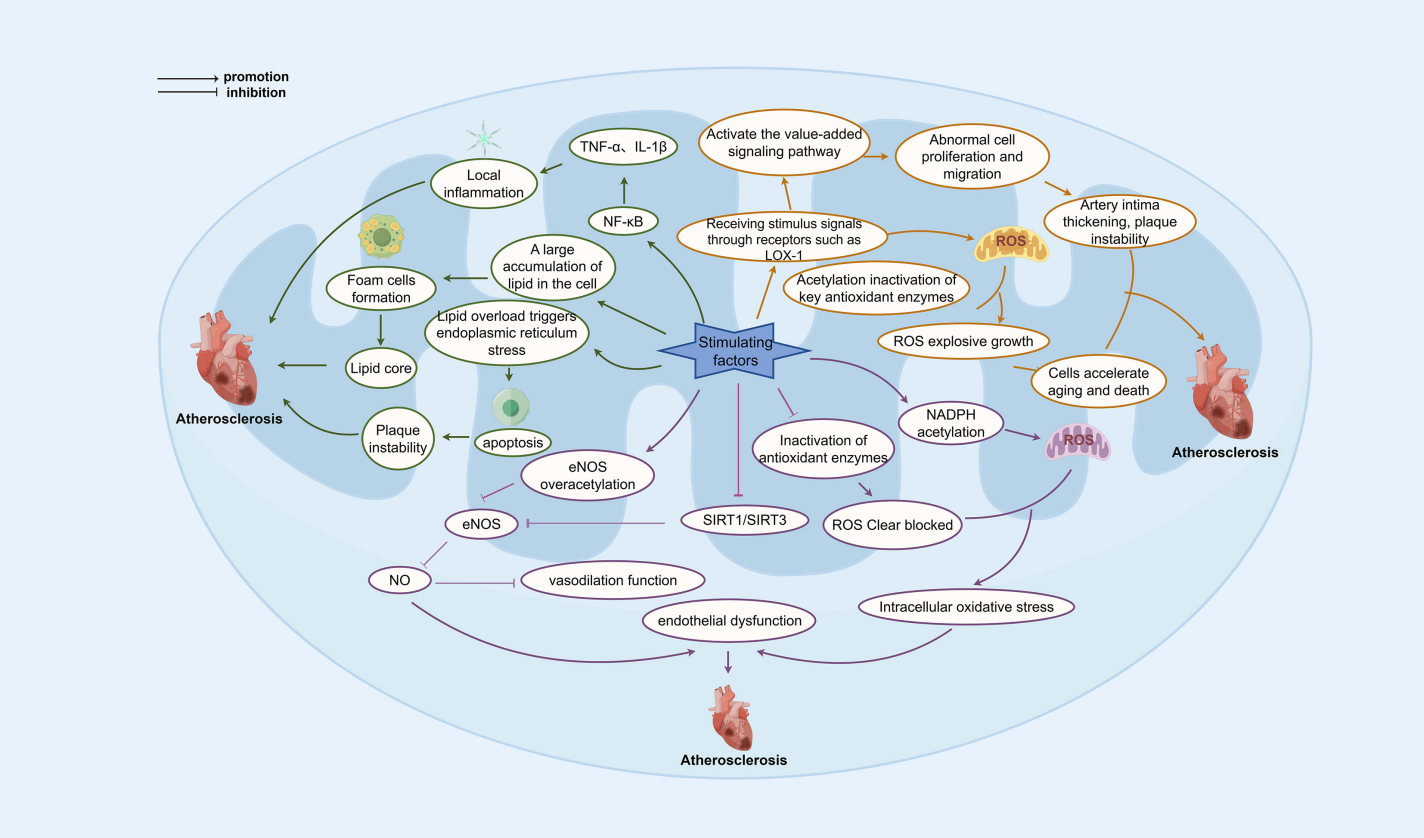
Glucose and lipid metabolism.

Protein acetylation modulates oxidative stress by regulating the balance between pro-oxidant and antioxidant systems, thereby contributing to AS progression. The pathogenesis of AS involves not only vascular lipid deposition and inflammatory responses but also hepatic lipid metabolic disturbances and oxidative stress (116). Persistent stimulation promotes continuous ROS generation, and excessive ROS accumulation triggers oxidative stress, ultimately leading to cellular and tissue injury (117).

In the vascular wall, several ROS-generating enzyme systems contribute to the pro-oxidative state. Acetylation of NADPH oxidase family members, such as NOX2, promotes subunit assembly and enzymatic activation, whereas acetylation of xanthine oxidase directly enhances its catalytic activity (118). These modifications collectively promote substantial ROS production (119). Conversely, acetylation of antioxidant enzymes, including superoxide dismutase (SOD), glutathione peroxidase (GPx), and catalase (CAT), may alter active-site conformation, weaken substrate binding, reduce protein stability, increase susceptibility to degradation, or disrupt subcellular localization, thereby impairing antioxidant defense capacity (120, 121).

Protein acetylation also regulates nitric oxide (NO) synthesis and release. NO is a major vasoactive mediator produced by vascular endothelial cells and exerts vasodilatory, antiplatelet, and anti-adhesive effects (122). eNOS is the key enzyme responsible for NO production, and its activity directly affects endothelial function (123). eNOS is regulated by several post-translational modifications, including acetylation, nitrosylation, and phosphorylation, with effects that depend on the modification site and cellular context. Evidence suggests that advanced glycation end products (AGEs) enhance AMPK phosphorylation, thereby stimulating SIRT1-mediated eNOS deacetylation. This process increases NO bioavailability and alleviates vascular dysfunction associated with dyslipidemia (124).

Endothelial cells form a monolayer lining the inner surface of blood vessels and act as a critical barrier for maintaining vascular homeostasis through the production of vasoactive mediators (122). Under physiological conditions, they preserve vascular tone by releasing NO, prostacyclin (PGI2), and endothelium-derived relaxing factor (EDRF) (125). The acetylation status of eNOS directly affects its interaction with calcium ions and calmodulin, thereby regulating enzymatic activity. Excessive eNOS acetylation suppresses its activity, reduces NO production, and impairs vasodilation (123). In contrast, SIRT1 and SIRT3 promote eNOS deacetylation, enhance NO synthesis, and improve endothelium-dependent vasorelaxation. Consistently, overexpression of SIRT1 or SIRT3 markedly increases eNOS activity and NO release, whereas their inhibition elevates eNOS acetylation, decreases NO production, and weakens vasodilatory capacity (126, 127).

Acetylated low-density lipoprotein (acetyl-LDL) stimulates VSMC proliferation and represents an important mechanism in AS progression (128). Studies have shown that resistin and acetyl-LDL induce abnormal proliferation and migration of human coronary artery smooth muscle cells (HCASMCs) while increasing intracellular ROS production. Resistin further suppresses mitochondrial SOD activity, thereby aggravating oxidative stress and cellular dysfunction (129). Notably, ginsenoside Rb1 attenuates these effects by reducing HCASMC proliferation and migration, lowering ROS levels, and restoring mitochondrial SOD activity. By rebalancing oxidative and antioxidant signaling, ginsenoside Rb1 may help preserve smooth muscle cell function and represents a potential therapeutic strategy for vascular injury and cardiovascular disease (128).

In summary, protein acetylation is deeply involved in AS initiation and progression by bidirectionally regulating pro-oxidant systems, including NOX and xanthine oxidase, and antioxidant systems, including SOD and GPx, as well as through site-specific modulation of eNOS activity and acetyl-LDL-associated smooth muscle cell proliferation. However, several limitations remain. First, mechanistic integration is still insufficient, as most studies focus on individual proteins or signaling pathways rather than systematically addressing the interconnected network of acetylation, oxidative stress, metabolism, and inflammation, as well as coordinated interactions among endothelial cells, VSMCs, and macrophages. Second, causal and dynamic evidence remains limited, making it difficult to determine whether acetylation changes are drivers or consequences of oxidative stress; moreover, dynamic profiling of acetylation across different stages of AS is still lacking. Third, translational evidence remains inadequate, because currently available deacetylase modulators often lack specificity, and precision strategies targeting key acetylation sites are still underdeveloped. Future studies should integrate single-cell technologies, epigenetic editing, and high-resolution proteomics to define the dynamic causal networks of acetylation and facilitate the development of site-specific therapeutic interventions for clinical translation.

### 4.4 Energy metabolism

**Figure 4.**

**Figure 4:**
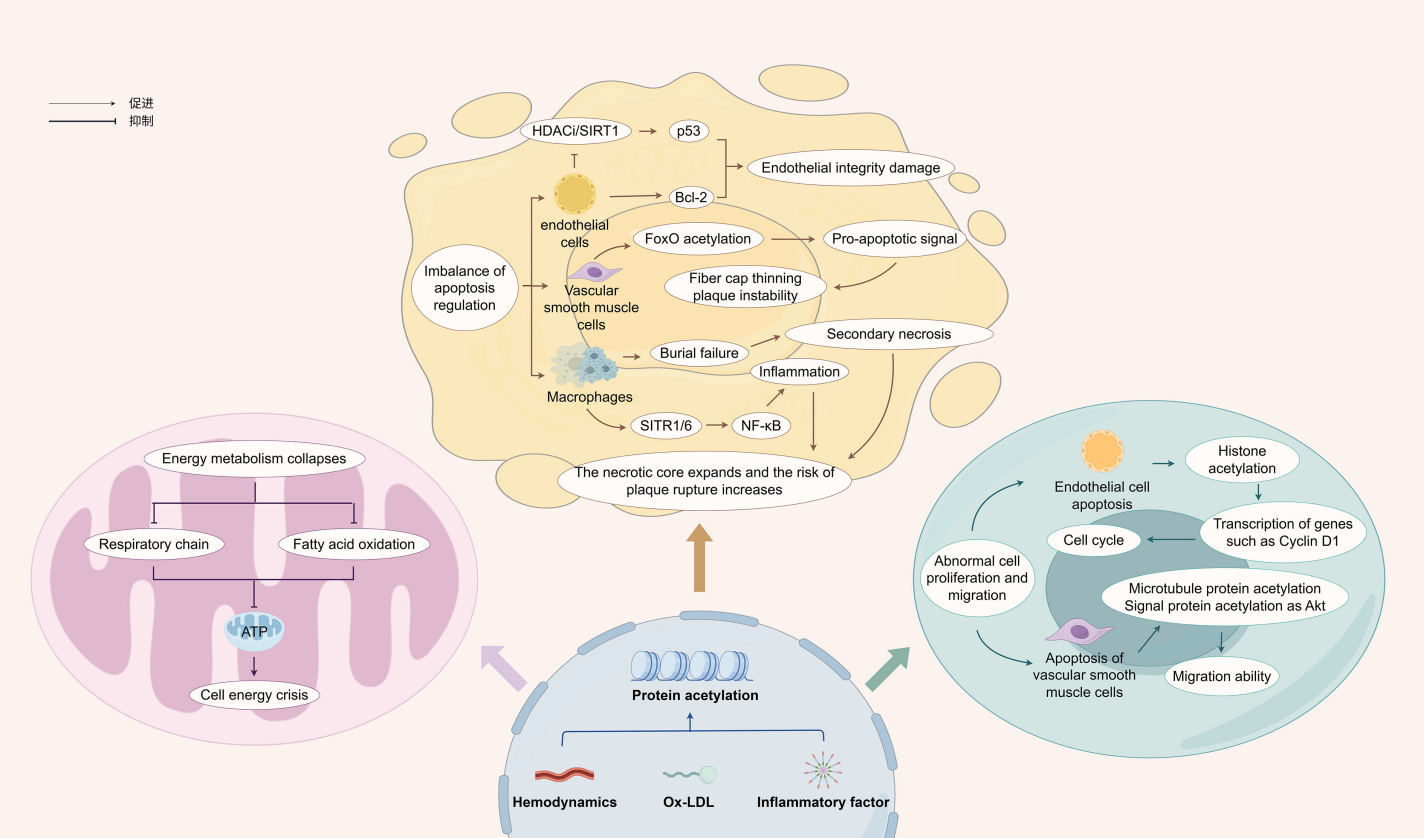
The roles of protein acetylation in energy metabolism, cell apoptosis, cell migration, and proliferation in relevant cells during atherosclerosis.

Cellular energy metabolism is essential for maintaining normal physiological function. As an important post-translational modification, protein acetylation regulates energy metabolism at multiple levels and enables cells to adapt to changes in energy status (130).

In AS, vascular cells exposed to pathogenic stimuli exhibit reduced expression of the mitochondrial deacetylase SIRT3, leading to excessive acetylation of mitochondrial respiratory chain proteins and subsequent functional impairment (131). This abnormal acetylation disrupts the activity of multiple respiratory chain complexes. Impaired complex I (CI) function reduces NADH oxidation and ATP production, whereas decreased activity of complex III (CIII) and complex IV (CIV) compromises electron transport efficiency. As a result, VSMCs and macrophages develop substantial defects in mitochondrial respiration that fail to meet cellular metabolic demands (132).

Proteomic analyses have revealed widespread acetylation of multiple subunits within mitochondrial respiratory chain complexes I–IV (121, 133). Among these, the acetylation-inhibition mechanism for CI is the best defined. Studies have shown that SIRT3 deficiency causes excessive acetylation of CI subunits (e.g., NDUFA9), markedly reducing NADH binding, electron transfer capacity, and proton-pumping capacity (134). CIV is also regulated by acetylation, and acetylation of components such as cytochrome b (MT-CYB) and COX5A has been associated with impaired complex assembly and activity under conditions of SIRT3 deficiency (135). In addition to oxidative phosphorylation, acetylation also regulates fatty acid oxidation. Carnitine palmitoyltransferase I (CPT-I), the rate-limiting enzyme for mitochondrial fatty acid uptake, is directly modulated by acetylation, and CPT1A has been identified as an important mitochondrial acetylation target (133). Several enzymes involved in mitochondrial β-oxidation, including acyl-CoA dehydrogenase, hydratase, hydroxyacyl-CoA dehydrogenase, and ketothiolase, also undergo dynamic acetylation. Notably, SIRT3-mediated deacetylation of long-chain acyl-CoA dehydrogenase (LCAD) is a key step in its activation (136). More broadly, acetylation appears to suppress enzymatic activity by altering the charge environment and conformational stability of catalytic domains (137).

In summary, current evidence suggests that SIRT3 downregulation and the resulting hyperacetylation of mitochondrial respiratory chain components and fatty acid oxidation enzymes are key mechanisms underlying energy metabolic dysfunction in vascular cells during AS. However, several important limitations remain. First, current studies have focused predominantly on SIRT3, whereas the roles of other acetyltransferases and deacetylases remain insufficiently explored. Second, functional analysis of specific acetylation sites is still limited, as many targets identified by omics approaches have not been rigorously validated by site-directed mutagenesis and mechanistic investigation. Future studies should emphasize precise mechanistic dissection, dynamic monitoring, and systematic integration of acetylation-dependent metabolic regulation to provide a stronger basis for therapeutic strategies targeting metabolic remodeling in AS.

### 4.5 Apoptosis

**Figure 4**.

Endothelial cells form the primary barrier of the vascular wall, and their apoptosis is considered a critical initiating event in AS. Under stimulation by risk factors such as disturbed hemodynamics, ox-LDL, and inflammatory mediators, endothelial dysfunction disrupts intracellular acetylation homeostasis (138). This balance, maintained by acetyltransferases and deacetylases, is closely linked to apoptotic regulation. HDAC inhibitors increase histone acetylation, including H3ac and H4ac, thereby opening chromatin structure, activating genes such as p21 and p53, promoting cell cycle arrest, and enhancing the expression of apoptosis-related genes (139). Among non-histone targets, p53 is a major substrate of SIRT1 and a central regulator of apoptosis-related transcription (140). The balance between pro-apoptotic Bcl-2 family members (e.g., Bax, Bak) and anti-apoptotic proteins (e.g., Bcl-2, Bcl-XL) is also influenced by acetylation-dependent signaling. In addition, HDAC inhibitors can enhance acetylation of p53, STAT3, and tubulin, thereby affecting multiple apoptotic pathways (138). Endothelial apoptosis compromises barrier integrity, increases vascular permeability, and facilitates lipid deposition and monocyte infiltration into the subendothelial space, representing an early and pivotal step in AS development.

VSMCs are the major cellular component of the arterial media, and their apoptosis has important consequences for plaque stability. In early AS, excessive VSMC apoptosis weakens vascular wall integrity, promotes lipid core formation, and destabilizes plaques (141, 142). Under oxidative stress and inflammatory cytokine stimulation, abnormal expression or activity of HDACs in VSMCs influences the expression of pro-apoptotic genes (143). Non-histone acetylation is also critically involved. For example, acetylation of forkhead box O (FoxO) transcription factors affects the regulation of downstream targets such as Bim and Fas ligand (FasL), thereby influencing cell fate decisions (144). As survival signaling declines and apoptotic pathways intensify, VSMC apoptosis contributes to fibrous cap thinning, reduced ECM synthesis, and increased plaque vulnerability (145, 146).

Macrophage apoptosis exerts stage-dependent effects during plaque development. In early lesions, apoptotic macrophages can be efficiently recognized and cleared by efferocytosis, thereby preventing the release of intracellular contents that would otherwise trigger inflammation and simultaneously promoting anti-inflammatory and tissue repair responses (147, 148). In advanced plaques, phagocytic efficiency declines, leading to delayed clearance of apoptotic cells. Consequently, secondary necrosis occurs, releasing damage-associated molecular patterns (DAMPs), amplifying local inflammation, enlarging the necrotic core, and promoting plaque instability (149, 150). Acetylation plays an important regulatory role in this process. Deacetylases such as SIRT1 and SIRT6 exert anti-inflammatory and anti-apoptotic effects. SIRT1 deacetylates NF-κB p65, suppresses its transcriptional activity, and reduces the production of pro-inflammatory cytokines, including TNF-α and IL-1β, thereby creating a less pro-apoptotic microenvironment (62, 151, 152). Conversely, inhibition of deacetylase activity activates both inflammatory and apoptotic pathways (153). Acetylation also influences macrophage polarization, with pro-inflammatory M1 macrophages generally showing greater susceptibility to apoptosis, whereas anti-inflammatory M2 macrophages are relatively more resistant (154). Thus, modulating acetylation to balance macrophage survival and death is essential for determining plaque progression and stability.

In summary, apoptosis of endothelial cells, VSMCs, and macrophages plays distinct yet interconnected roles in AS progression. Endothelial apoptosis initiates barrier disruption, VSMC apoptosis weakens fibrous cap integrity, and macrophage apoptosis exerts stage-dependent effects that are protective in early lesions but detrimental in advanced plaques when efferocytosis fails. Acetylation acts as a central regulatory mechanism throughout these processes: histone acetylation promotes chromatin relaxation and apoptosis-related transcription, whereas non-histone acetylation of proteins such as p53, FoxO, and NF-κB fine-tunes the magnitude and direction of apoptotic signaling. However, current evidence is derived largely from *in vitro* studies and animal models, and direct analysis of the temporal relationship between acetylation dynamics and apoptosis at single-cell resolution in human tissues remains limited. In addition, mechanistic studies have focused mainly on a few classical targets, whereas non-histone acetylation networks, compensatory interactions among deacetylases, and crosstalk with other post-translational modifications remain insufficiently understood. The inconsistent effects of HDAC inhibitors in AS further highlight the lack of stratified studies by disease stage and cell type, which currently limits clinical translation. Future studies should integrate human tissue samples with spatial and single-cell technologies to construct a spatiotemporal atlas of the acetylation–apoptosis network and to support the development of cell-specific and stage-dependent acetylation-targeted precision therapies.

### 4.6 Cell proliferation and migration

**Figure 4**.

Protein acetylation plays an important role in regulating VSMC proliferation and migration, two key processes involved in atherosclerotic remodeling. Histone acetylation strongly influences the transcription of genes associated with VSMC proliferation. For example, cyclin D1, a critical regulator of the G1/S phase transition, is upregulated during VSMC proliferation. Increased histone acetylation enhances transcription factor access to the promoter regions of proliferation-related genes, including cyclin D1, thereby promoting their transcription and expression (155–157). PDGF has been shown to induce histone acetylation and chromatin remodeling, facilitating the binding of transcription factors such as serum response factor (SRF) to regulatory regions of genes involved in VSMC proliferation (158). Non-histone acetylation also contributes to the regulation of VSMC proliferative activity.

Protein acetylation further influences VSMC migration by modulating cytoskeletal organization, adhesion molecule expression, and intracellular signaling pathways (155). HDAC6, a major microtubule deacetylase, targets the K40 residue of α-tubulin. Reduced HDAC6 activity or pharmacologic inhibition increases tubulin acetylation, stabilizes microtubules, and promotes VSMC migration (159, 160). Histone acetylation also regulates transcriptional programs that control migration-related genes, including adhesion molecules, thereby influencing VSMC motility and vascular lesion progression (155). Acetylation of the signaling protein Akt directly modulates its kinase activity and downstream pathways, further affecting VSMC migration (161).

However, several limitations remain. Current studies have paid insufficient attention to the heterogeneous responses of distinct VSMC subpopulations, and supporting single-cell evidence is still limited. In addition, most available data are derived from *in vitro* experiments, whereas cell-specific causal relationships *in vivo* remain to be established using approaches such as lineage tracing and conditional gene editing. Future studies should therefore focus on dynamic epigenetic regulatory networks, cellular heterogeneity, and precision intervention strategies.

### 4.7 Dysregulated acetylation in human AS

Although animal models have provided important *in vivo* evidence for elucidating how acetylation regulates AS, studies based on human samples are essential for bridging basic research and clinical translation. Increasing evidence indicates that protein acetylation is markedly dysregulated in human atherosclerotic lesions (112). Among acetylation-related regulators, p300 exerts pro-atherogenic effects by acetylating transcription factors such as NF-κB, thereby promoting inflammatory gene expression and macrophage foam cell formation (63). In contrast, class III histone deacetylases, particularly SIRT1 and SIRT6, display clear atheroprotective properties and are downregulated in human plaques (64, 65). By deacetylating both histone and non-histone substrates, sirtuins suppress inflammation, preserve endothelial function, and promote reverse cholesterol transport. In addition, altered expression of other deacetylases, including HDAC3 and HDAC9, has also been reported in human plaques, and increased HDAC9 expression has been associated with AS susceptibility in genome-wide association studies (162, 163). Human-based studies have further supported the pathological relevance of acetylation-regulated metabolic signaling. Acetate, after conversion to acetyl-CoA by ACSS2, promotes acetylation of the TGF-β receptor ALK5 and SMAD2/4, forming a positive feedback loop that drives EndMT and accelerates atherosclerotic progression (80). Inhibition of ACSS2 disrupts this pathway and alleviates AS. Importantly, studies using human aortic tissues and human-derived endothelial cells have confirmed that ACSS2 is highly expressed in human atherosclerotic lesions and that the acetate–acetylation axis promotes EndMT in human cells, consistent with findings from murine models. Collectively, these observations indicate that disruption of the acetylation-deacetylation balance is a key epigenetic feature of human AS and contributes to disease initiation and progression by regulating inflammation, lipid metabolism, and endothelial dysfunction.

## 5 Therapeutic approaches

**Table 1**.

**Table 1:** Therapeutic agents targeting protein acetylation in atherosclerosis.

Category	Key Targets/Subtypes	Core Mechanisms	Representative Drugs/Substances	Main Anti-AS Effects	Features& challenges	References
Histone Acetyltransferases (HATs)	p300/CBPCrebbp/Ep300/Kat2a/Kat2b	Overactivation promotes pro-atherogenic gene expression, NF-κB activation, vascular inflammation, and foam cell formation; inhibition blocks these pathological pathways	C646, A-485, Curcumin, EGCG, Itaconate (ITA-LNP)	Inhibits endothelial inflammation, reduces macrophage foam cell formation, decreases IL-6/TNF-α, suppresses inflammatory gene transcription	Good inhibitor selectivity; natural products show multi-target effects and low specificity	(164–171)
Histone Deacetylases (HDACs)	Pan-HDAC	Non-selective HDAC inhibition globally increases acetylation levels	Trichostatin A (TSA)	No anti-AS benefit; aggravates atherosclerotic lesions	High risk of non-selective inhibition, exacerbates AS	(172)
	Class I HDACs (HDAC1/2/3)	Inhibits HDAC1/2/3 → upregulates anti-inflammatory IL-10 and suppresses pro-inflammatory genes	Valproate	Alleviates AS in hyperglycemic ApoE⁻/⁻ mice	Favorable subtype selectivity, clear anti-inflammatory effects	(173)
	Class II HDACs (HDAC5/6, etc.)	Inhibits tubulin deacetylation → reduces VSMC migration and proliferation	Tubastatin A	Enhances plaque stability	Focuses on plaque stability regulation	(174)
	Class IV HDAC	Improves tissue injury and inhibits NLRP3 inflammasome activation	Entinostat	Improves inflammation and tissue damage; direct anti-AS effects remain to be studied	Insufficient direct evidence for anti-AS efficacy	(175)
	HDAC2/3	Inhibits HDAC activity, increases histone acetylation, activates NRF2 antioxidant pathway	Sulforaphane	Antioxidative stress, improves endothelial function and metabolism	Natural origin, dual HDAC inhibition and antioxidant effects	(176, 177)
	HDAC11	Statins inhibit HDAC11 → upregulate EphA2 → aggravate inflammation; targeting this axis reverses side effects	Statins+EphA2 inhibitors	Counteracts pro-inflammatory side effects of statins and exerts synergistic anti-AS effects	Reveals acetylation-related adverse effects of statins; provides combination strategy	(178)
Sirtuins and Their Activators	SIRT1	Activation deacetylates eNOS and NF-κB → improves endothelial function and suppresses inflammation	Astaxanthin, Resveratrol, SRT1720	Enhances NO bioavailability, inhibits inflammation, reduces lipid accumulation, delays AS progression	Activator-based, well-defined mechanisms	(179–181)
	SIRT3	Activation regulates endothelial metabolism, lipid metabolism, and angiogenesis	Resveratrol, Ginsenosides	Prevents vascular disorders and reduces AS risk	Multi-pathway vascular protection	(182)
	SIRT7	Upregulates Sirt7 → deacetylates Keap1 → activates Nrf2 antioxidant pathway	Ganoderma lucidum spore powder	Delays vascular senescence,alleviates AS and vascular calcification	Natural product; incompletely validated mechanisms, multi-target uncertainty	(183)
Overall Status & Challenges	—	Targets acetylation homeostasis to intervene in AS via inflammation, metabolism, oxidative stress, and cellular functions	—	Anti-inflammatory,lipid-regulating, plaque-stabilizing,endothelial-protective	No drugs officially approved for AS; core limitations: lack of tissue specificity, poor long-term safety, off-target effects	
Future Directions	—	Improve subtype selectivity, develop vascular/macrophage-targeted delivery, optimize low-dose long-term regimens	—	Balance efficacy and safety to accelerate clinical translation	Breakthroughs in selectivity,targeted delivery,and long-term safety for precision therapy	

In recent years, therapeutic strategies targeting protein acetylation have emerged as a promising therapeutic direction in AS research. The central concept is to restore intracellular acetylation homeostasis by modulating the activity of three major enzyme families, including HATs, HDACs, and sirtuins, thereby intervening in multiple pathological processes involved in AS. However, despite their therapeutic promise, current strategies still face major translational challenges, as their efficacy is highly dependent on specific targets, cell types, and disease stage.

Among acetylation-related targets, the histone acetyltransferase p300 has attracted particular attention because it promotes pro-atherogenic gene expression through acetylation of both histone and non-histone proteins, including NF-κB, thereby enhancing vascular inflammation, macrophage foam cell formation, and disease progression. Accordingly, p300 inhibition may suppress these pathological pathways. Multiple HDAC isoforms have also been implicated in AS. For example, HDAC3 inhibits cholesterol efflux in macrophages, HDAC5 impairs endothelial function, HDAC9 exacerbates plaque inflammation, and HDAC6 promotes endothelial oxidative injury. Targeted inhibition of these isoforms may therefore improve cholesterol metabolism and activate anti-inflammatory or vasculoprotective pathways. In contrast, several sirtuins, particularly SIRT1, SIRT2, and SIRT6, exert atheroprotective effects by maintaining vascular cell homeostasis, suppressing inflammation, inhibiting foam cell formation, and ameliorating lipid metabolic disturbances through deacetylation of key substrates. Therefore, activation of these enzymes has been proposed as a promising anti-atherosclerotic strategy.

### 5.1 Histone acetyltransferases

Among HATs, p300 and CREB-binding protein (CBP) are key members closely associated with AS, and their hyperactivation may be linked to pro-inflammatory gene expression (184). Selective small-molecule inhibitors of p300/CBP, such as C646 and A-485, have shown promising effects in preclinical studies by suppressing inflammatory responses in vascular endothelial cells and reducing macrophage foam cell formation (164, 165). Curcumin, which also exhibits inhibitory activity against p300/CBP-associated HAT function, has been reported to reverse the hyperglycemia-induced increase in HAT activity and decrease in HDAC activity in THP-1 monocytes, thereby reducing NF-κB transcriptional activity and lowering the production of IL-6 and TNF-α (166–168). In animal models, curcumin supplementation ameliorated high-fat diet-induced insulin resistance and hyperglycemia, and these protective effects were partly attributed to its anti-inflammatory activity (169). Epigallocatechin gallate (EGCG) has also been identified as a potent HAT inhibitor. By suppressing HAT activity, EGCG reduces NF-κB acetylation, attenuates its transcriptional activity, and decreases p300 recruitment to the promoters of pro-inflammatory genes such as IL-6 (170). Recent studies have shown that IRG1/itaconate is highly expressed in unstable plaques but remains relatively low in stable plaques (171). Mechanistically, itaconate suppresses inflammatory gene transcription by inhibiting the activity of multiple HATs, including CREBBP, EP300, KAT2A, and KAT2B, and by reducing H3K27 acetylation. To overcome the delivery limitations of itaconate, an ITA-LNP nanodelivery system was developed to enable targeted delivery to plaques and bone marrow. This strategy also showed a favorable safety profile, with no evident hepatotoxicity, supporting its translational potential. In addition, the inclusion of human stable and unstable plaque samples provided further translational evidence by confirming differential IRG1 expression in human lesions. Notably, the reported differences in efficacy between unmodified itaconate and its derivatives further highlight the importance of carefully evaluating compound-specific effects, target selectivity, and potential off-target actions.

### 5.2 Histone deacetylases

HDACs have emerged as promising therapeutic targets in atherosclerosis (AS). Inhibition of HDAC activity increases the acetylation levels of histones and other functional proteins, including transcription factors and inflammatory signaling molecules, thereby modulating gene expression networks and exerting anti-atherosclerotic effects. Research on HDAC inhibitors (HDACis) targeting different isoforms has progressed from preclinical studies to early-stage clinical exploration.

According to their chemical structure and isoform selectivity, HDAC-targeting agents can be broadly classified as pan-HDAC inhibitors, class I-selective inhibitors, class II-selective inhibitors, sirtuin modulators, and class IV HDAC inhibitors (185). However, their therapeutic effects depend strongly on target selectivity. Non-selective HDAC inhibitors may produce unfavorable outcomes. For example, trichostatin A (TSA) has been reported to aggravate atherosclerotic lesion formation in Ldlr^-^/^-^ mice, highlighting the potential risks of broad-spectrum HDAC inhibition (172). Sulforaphane, another HDAC inhibitor, has been shown in cellular and animal studies to suppress HDAC activity, particularly by reducing HDAC2 and HDAC3 protein levels, while increasing histone H3 and H4 acetylation. In addition, HDAC inhibition by sulforaphane may activate the nuclear factor erythroid 2-related factor 2 (NRF2) pathway, a central regulator of antioxidant defense (176, 177). In contrast, selective HDAC inhibitors appear to have more favorable anti-atherosclerotic effects. The class I inhibitor valproate attenuates AS in hyperglycemic ApoE-deficient mice by inhibiting nuclear HDAC1/2/3, increasing IL-10 expression, and suppressing pro-inflammatory gene transcription (173, 186). The class II inhibitor Tubastatin A improves plaque stability by inhibiting cytoplasmic tubulin deacetylation and reducing VSMC proliferation and migration (174). The class IV inhibitor entinostat has also been shown to alleviate histopathological injury, local inflammation, and NLRP3 inflammasome activation, although its direct effects in AS require further investigation. Furthermore, emerging evidence indicates that statins may inhibit HDAC11, thereby reducing KLF4 binding to the EphA2 promoter and increasing histone H3/H4 acetylation at this locus. This change promotes EphA2 expression and activates the NLRP3/NF-κB signaling pathway, leading to enhanced macrophage inflammatory responses. Importantly, inhibition of EphA2 within this acetylation-regulated axis may mitigate the pro-inflammatory effects of statins and synergize with statin therapy to enhance anti-atherosclerotic efficacy (175, 178). These findings highlight the importance of acetylation in drug-related adverse effects and suggest a potential combination strategy for optimizing statin-based therapy. They also raise the possibility that interindividual variability in the HDAC11/EphA2 axis may influence therapeutic responses in clinical settings.

### 5.3 Sirtuin-targeted therapeutic strategies

Increasing evidence suggests that activation of sirtuins represents a promising therapeutic strategy for AS. Astaxanthin (ASTX), for example, has been reported to activate peroxisome proliferator-activated receptor alpha (PPARα) while inhibiting peroxisome proliferator-activated receptor gamma (PPARγ) and protein kinase B (Akt) signaling, thereby reducing hepatic lipid accumulation. In addition, ASTX suppresses nuclear translocation of SREBP-1, reduces lipid synthesis, and alleviates lipid deposition through regulation of autophagy-related pathways. These effects appear to be closely associated with SIRT1-mediated deacetylation and may collectively contribute to slowing atherosclerotic progression (187, 188, 189). SIRT1 activators such as resveratrol and SRT1720 have also shown anti-atherosclerotic potential by increasing nitric oxide bioavailability, improving endothelial function, and suppressing inflammatory signaling through deacetylation of eNOS and NF-κB (189). In addition to SIRT1, SIRT3 has emerged as an important regulator of vascular homeostasis. Resveratrol and ginsenosides have been reported to attenuate vascular injury and reduce atherosclerotic risk by modulating endothelial metabolism, lipid metabolism, and angiogenesis through SIRT3 activation (179, 190). More recently, Ganoderma lucidum spore powder has been shown to upregulate SIRT7 expression, promote Keap1 deacetylation, and facilitate dissociation of Keap1 from Nrf2, thereby activating the Nrf2 antioxidant pathway. Through this mechanism, it may delay vascular aging and alleviate both AS and vascular calcification (180, 181). Collectively, these findings suggest that pharmacological activation of specific sirtuin isoforms may simultaneously modulate inflammation, metabolism, oxidative stress, and vascular cell function, offering a potentially more refined therapeutic strategy than non-selective deacetylase modulation. Nevertheless, the development of sirtuin-targeted interventions still faces several challenges. For natural compounds in particular, mechanistic interpretation is often complicated by multitarget effects and limited specificity. For example, it remains unclear whether the antioxidant activity of Ganoderma lucidum spore powder depends primarily on SIRT7-mediated Keap1 deacetylation or also involves additional signaling pathways, which will require further validation through gene knockout and other mechanistic approaches. In addition, the active components, bioavailability, pharmacokinetics, and dose-response relationships of many natural products remain insufficiently defined, limiting their translational potential.

Currently, most deacetylase-targeted strategies for AS remain at the preclinical stage. Although several compounds have shown encouraging effects in experimental models, major barriers to clinical translation persist, including limited tissue specificity and concerns regarding long-term safety. Because deacetylases are widely expressed, systemic modulation may lead to unintended off-target effects. Future studies should therefore prioritize the development of isoform-selective agents, targeted delivery systems directed toward vascular tissues or macrophages, and low-dose long-term regimens better suited to chronic disease management.

In summary, targeting sirtuin-dependent deacetylation represents a promising epigenetic strategy for AS. Although no sirtuin-based therapy has yet been approved for this indication, accumulating preclinical evidence indicates that activation of specific sirtuins can exert anti-inflammatory, antioxidant, endothelial-protective, and lipid-regulatory effects. Further advances in isoform selectivity, tissue targeting, and long-term safety evaluation will be essential for translating these findings into precision therapies for AS.

## 6 Clinical application limitations of protein acetylation-targeted therapies in AS

The HDAC family comprises 18 members, and their functions in AS are highly dependent on both isoform and cell type, with some isoforms even exerting opposing effects. For example, HDAC9 promotes inflammation and foam cell formation in macrophages, yet may contribute to plaque stability in VSMCs by restraining excessive proliferation (162, 163). This functional heterogeneity presents a fundamental obstacle to the rational design of selective HDAC-targeted therapies. In addition, non-selective HDAC inhibitors, such as TSA, have been shown to aggravate atherosclerotic lesions in Ldlr-/- mouse models, whereas pan-HDAC inhibitors used in oncology have been associated with serious adverse effects, including thrombocytopenia, QT interval prolongation, and cardiotoxicity (182). These toxicities are particularly problematic in patients with AS, who often require long-term treatment. The therapeutic development of sirtuin activators remains controversial. The proposal that resveratrol directly activates SIRT1 has been increasingly questioned, and later studies suggest that its effects may instead be mediated indirectly through phosphodiesterase inhibition. Furthermore, the clinical development of SIRT1 activators such as SRT1720 has been halted because of insufficient efficacy or safety concerns. Together with the potential tumor-promoting risks of long-term systemic SIRT1 activation and the technical challenges of selectively targeting mitochondrial sirtuins with small molecules, these issues continue to limit the clinical translation of sirtuin-based therapies (183).

AS is fundamentally a localized vascular disease, whereas most currently available agents are administered systemically, creating an inherent mismatch between inadequate drug accumulation within plaques and the risk of systemic toxicity. Broad inhibition of HDAC activity may also impair normal immune surveillance, thereby increasing the risk of infection or autoimmune-like adverse reactions. Although nanoparticle-based plaque-targeted delivery systems represent a promising strategy to overcome this limitation, their clinical translation remains constrained by several technical and safety challenges, including manufacturing complexity, limited targeting efficiency, and concerns regarding biocompatibility (171). Moreover, disease heterogeneity presents a major challenge for defining effective therapeutic windows. Acetylation may exert markedly different roles in early versus advanced plaques, and considerable interindividual variability exists in human lesions. In contrast, most preclinical studies rely on genetically homogeneous animal models, which fail to adequately reflect the complexity of human disease and may contribute to the poor translation of preclinical findings into clinical benefit. To date, no drugs targeting acetylation-modifying enzymes have been approved as first-line therapies for AS. Future progress will depend on the development of combination strategies integrated with statin therapy and on precise patient stratification based on plaque acetylation signatures or genetic background.

## 7 Conclusion

AS is a chronic inflammatory disease driven by the complex interplay of multiple cell types, molecular mediators, and signaling pathways throughout its initiation and progression. Current evidence indicates that targeting protein acetylation-modifying enzymes represents a promising therapeutic strategy for AS. However, available studies also show that different classes of HDACs, and even distinct isoforms within the same class, can exert divergent effects in AS. Pan-HDAC inhibitors (e.g., TSA) may exacerbate lesions due to insufficient specificity, whereas selective inhibitors (e.g., class I HDAC inhibitors such as valproate) or sirtuin activators exhibit therapeutic potential. Furthermore, nutrients and dietary bioactive compounds may modulate the activity of deacetylases such as SIRT1 and SIRT3, thereby exerting antioxidant, anti-inflammatory, and metabolic protective effects. These findings provide an epigenetic basis for integrating dietary and lifestyle interventions into anti-atherosclerotic strategies.

Despite substantial progress, several key questions remain unresolved. First, most current evidence is derived from whole-tissue analyses or *in vitro* cell models and cannot fully capture the cell-specific responses of macrophages, endothelial cells, and VSMCs to acetylation changes *in vivo*. Future studies should integrate single-cell multi-omics with cell type-specific experimental models to define these regulatory networks with greater precision. Second, much of the available evidence remains correlative. Approaches such as chemical genetics and conditional gene editing will be required to manipulate acetylation events within defined spatiotemporal contexts and thereby establish causal relationships. Third, clinical translation is still hindered by species differences, highlighting the need to construct acetylation profiles from human plaque samples and to validate associations between genetic variation and disease risk in large population-based cohorts. Finally, current pharmacological interventions, including HDAC inhibitors and sirtuin activators, remain limited by off-target effects and insufficient isoform selectivity. The development of next-generation agents with plaque-targeting capacity and high isoform specificity will be essential for overcoming these translational barriers and advancing precision therapy for AS.
